# Diagnosis of Inflammation of the Grey, and the White Matter of the Brain

**Published:** 1842-03

**Authors:** 


*Diagnosis of Inflammation of the grey, and the white matter of the Brain.*—Prof. Bellengeri gives the following diagnostics to enable us to decide what part of the brain is inflamed. The white matter is the organ of motion, the grey of sensation and intellect—hence when the grey matter is irritated or inflamed, the intellectual faculties are disturbed with loquacity, and delirium; the senses, especially those of touch, are dull. Cerebral congestion in consequence of the compression it produces upon this substance, produces numbness and somnolency. When the white matter is affected, there is a lesion of the motive apparatus, spasms or paralysis, especially if the optic thalami, the corpora striata, or the cerebellum is attacked.

If the disease is not intense there is spasm, but if it is, then paralysis occurs. Spasms of the flexor muscles (*emprosthotonos*), indicates an affection of the cerebral hemispheres, spasm of the extensors (*opisthotonos*), one of the cerebellum. When the senses and the motor apparatus are alike affected, we should conclude that the lesion extended to both the white and grey matter. Diseases of the spinal marrow easily affect the motion because the white matter abounds there. To prove this fact the Professor relies upon the cases of Royer, Collard, Bayle, Rullier, &c. He believes, contrary to the opinion of Bell, that the posterior columns regulate motion, the anterior sensation. Simple pain in the head, particularly when severe, and not accompanied by delirium, torpor or spasm, indicates a lesion confined to the membranes, the dura mater, pia mater or arachnoid; encephalitis, accompanied by delirium, indicates that inflammation is seated in the grey matter of the brain, especially on the periphery of the organ. It is true that in encephalitis with delirium, whether acute or chronic, autopsy often reveals inflammation of the membranes, but the author believes that the delirium should not be attributed to the affection of the membranes, but to its being propagated to the grey matter of the substance of the brain, which is contiguous to the membranes.

As all the superficies whether external or internal, is covered with a pia mater, we cannot conceive of inflammation limited to the grey or the white matter, and not propagated to the membrane from which they derive their vessels. There cannot, therefore, be there a phlogosis of the cerebral substance without meningitis. In comatose or apoplectic encephalitis, the disease whether phlogosis or congestion, attacks the deeper seated portions of the grey matter; if it follow a phlogosis, its intensity indicates the severity of the disease and the need of further depletion, unless it depend on serous effusion which can be recognized by symptoms, and requires appropriate treatment. Convulsive, spasmodic or paralytic encephalitis, indicates an affection of the medullary (white) matter.—*N. Y. Med. Gaz. from the Jour. des Sciences Med. de Turin.*

